# Medical follow-up for workers exposed to bladder carcinogens: the French evidence-based and pragmatic statement

**DOI:** 10.1186/1471-2458-14-1155

**Published:** 2014-11-06

**Authors:** Bénédicte Clin, “RecoCancerProf” Working Group, Jean-Claude Pairon

**Affiliations:** Cancers and prevention, U1086 INSERM, Faculty of Medicine, Caen University Hospital, Caen, France; Service de Santé au Travail et Pathologie Professionnelle (Occupational Health Department), C.H.U. (University Hospital) Côte de Nacre, 14033 CAEN Cedex, France; INSERM, Unité 955, Université Paris-Est Créteil, 94000 Créteil, France; Centre Hospitalier Intercommunal de Créteil, Service de Pneumologie et de Pathologie Professionnelle, 94000 Créteil, France

**Keywords:** Bladder cancer, Occupational exposure, Medico-professional follow-up, Recommendations

## Abstract

**Background:**

The aim of this work was to establish recommendations for the medical follow-up of workers currently or previously exposed to carcinogenic substances for the bladder.

**Methods:**

A critical synthesis of the literature was conducted. Sectors of activity where workers are or were exposed to carcinogenic substances for the bladder were listed and classified according to the level of bladder cancer risk. Performances of techniques available for the targeted screening of bladder cancer were analysed, including a simulation of results among high-risk populations in France.

**Results:**

The risk level for the professional group and the latency period between the start of exposure and the natural history of the disease were selected to define a targeted screening protocol. The NMP22BC test, exclusive haematuria testing, and combinations of urine cytology with, respectively, the NMP22BC test and haematuria test, generated an extremely high proportion of false positive results.

**Conclusion:**

Urine cytology is the test that offers the best specificity. Although poor for all bladder cancer stages and grades combined, its sensitivity is better for high grades, which require early diagnosis since late-stage cancers are of very poor prognosis. These results suggest that urine cytology is currently the only technique suitable for proposal within the context of a first line targeted screening strategy for occupational bladder cancer. An algorithm summarising the recommended medical follow-up for workers currently or previously exposed to carcinogenic substances for the bladder is proposed, based on the level of risk of bladder cancer.

**Electronic supplementary material:**

The online version of this article (doi:10.1186/1471-2458-14-1155) contains supplementary material, which is available to authorized users.

## Background

With an estimated incidence of 386,000 cases of bladder cancer worldwide in 2008 and over 150,000 deaths, bladder cancer is the 9th cause of cancer in the world and the 8th cause of death by cancer in men [1, 2]. In France, bladder cancer is the 7th cause of cancer and the 8th cause of death by cancer in men, with an estimated incidence of 10,729 cases in 2009, causing over 4,500 deaths [3]. The annual incidence rates for this cancer in men and women in France were respectively 14.7/100000 and 2.5/100000 in 2012 [4].

Besides tobacco smoking, occupational exposure to carcinogens is another major risk factor for bladder cancer. Indeed, according to study results, the proportion of bladder cancers attributable to occupational exposure ranges from 5 to 25% in men [5–8]. In 2001, the InVS (Institut de Veille Sanitaire) estimated that, in France, 8 to 14% of incident cases of bladder cancer and 10 to 14% of deaths linked to this type of cancer in men were attributable to occupational exposure, representing 625 to 1,110 incident cases and 347 to 492 deaths by bladder cancer in 1999 [8]. The most frequent occupational sectors where excess rates of bladder cancer are observed are those exposing (or having exposed) workers to aromatic amines, nitrosamines and polycyclic aromatic hydrocarbons (PAHs). In France, even if measures have been taken to prohibit the use of carcinogens such as certain aromatic amines, and although preference has been given to the use of certain substitute products (e.g. bitumen instead of coal tar), certain industrial sectors remain implicated in exposure to bladder carcinogens (in particular, those using o-toluidine, o-tolidine, o-anisidine and MBOCA, and those using products containing secondary amines likely to react with nitrosating compounds). Priority must therefore be placed on implementing primary preventive measures on these sites.

The latency after the start of exposure to a carcinogenic substance for the bladder is estimated between 14 and 26 years [9] and appears rarely to fall under 20 years [10]. In the majority of cases, urothelial tumours appear after the age of 60 years [2, 11]. According to data from the international literature on the subject, bladder cancer screening in the general population is not recommended, due, in particular, to the disease’s low prevalence [12]. Furthermore, no study has been conducted relying on a sufficiently large sample of individuals to assess the relevance of bladder cancer screening in high-risk populations. Nevertheless, according to an international panel of experts (International Consensus Panel on Cytology and Bladder Tumour Markers), reunited in 2005, individual screening in high-risk subjects such as smokers, occupationally-exposed subjects and subjects with a genetic predisposition could be considered using, in particular, urinary makers associated or not with conventional cytology [13].

Data from the literature on our knowledge of certain occupational risk factors and dose–response relationships are as yet limited, as it is on bladder cancer screening tools and protocols. We therefore deemed absolutely necessary the compilation of a synthesis of available data, in order to determine associated strategy(ies) suitable for recommendation.

We deemed necessary to assess the efficiency of new techniques used in urology for the early diagnosis of bladder cancer recurrence, in order to rationalise medico-professional monitoring modalities for subjects presenting a high risk of bladder cancer.

The management of occupational carcinogenic risk for the bladder relies on both a technical preventive approach (i.e. the implementation of preventive measures, e.g. suppression or limitation of the exposure to occupational carcinogens) and on specific medical follow-up. We deliberately focus here on this second aspect. Indeed, the aim of this work was to define modalities for the medical follow-up of workers currently or previously exposed to carcinogenic substances for the bladder, which are adapted to exposure situations and coherent with current knowledge on dose–response relationships, evolutive characteristics and therapeutic options for the treatment of bladder cancer. We consequently defined target populations depending on risk levels and proof levels based on analysis of the scientific literature, together with the current more appropriate screening tool and frequency of screening.

## Methods

### Population concerned

These medical recommendations target all healthcare professionals involved in primary and secondary prevention of bladder cancer, to employers and to workers currently or previously exposed to carcinogenic substances for the bladder, whether they are still active or not and independently of their professional status.

Healthcare professionals include the occupational physician and the other members of the pluridisciplinary occupational health team (occupational health nurse and professionals involved in primary prevention of occupational risks) during the worker’s period of professional activity, then the general practitioner and/or urologist, or other healthcare professionals (e.g. in occupational disease consultation centres - within the context of post-occupational follow-up, oncology networks and anatomopathologists).

### Method

The subject of our study is vast and raises a number of questions and sub-questions. Available scientific data are highly dispersed and difficult to summarise; however, in principle, the subject does not require the initiation of a public debate. Furthermore, the most appropriate method appeared to be the RPC "Clinical Practice Guidelines" method, recommended by the HAS (French National Authority for Health). Analysis and critical synthesis of the scientific literature were conducted according to principles of critical reading, in order to attribute a level of scientific proof to each article, according to the classification recommended by the HAS (Table 1).

**Table 1:** **Recommendation grading (according to the "Guide d’analyse de la littérature et gradation des recommandations" - Literature analysis and recommendation grading guide, HAS, January 2000)**

Level of scientific proof provided by the literature (for clinical studies)	Recommendation grading
**Level 1**	
● High-power randomised comparative studies	**A**
● Meta-analysis of randomised comparative studies	Scientific proof established
● Decision analysis based on well-conducted studies	
**Level 2**	
● Low-power randomised comparative studies	**B**
● Well-conducted non-randomised comparative studies	Scientific proof presumed
**Level 3**	
● Case–control studies	
	**C**
**Level 4**	Low level of proof
● Comparative studies with major bias	
● Retrospective studies	
● Case series	

No randomised studies of occupational risk factors are usually conducted in the working environment. In contrast, there have been several "well-conducted" studies, taking into account confounding factors and potential dose–response relationships, together with a number of studies with concordant results. We consequently considered that meta-analysis or systematic reviews on well-conducted cohort studies offered level 1 scientific proof. We considered that cohort studies which were "well-conducted non-randomised studies" offered level 2 scientific proof, whereas, case–control studies were considered as offering level 3 scientific proof.

Due to a lack of available studies, recommendations are based on expert consensus within the framework of a work group after consultation with the reading group.

With regard to bladder cancer risk levels and the minimum exposure duration associated with a high risk of bladder cancer, when information was available, we agreed upon:

- a moderate relative risk for bladder cancer, for statistically significant relative risk (RR), odds ratio (OR) or standardised mortality ratio (SMR) observed in the scientific literature strictly above 1 and equal to or below 2;
- a high relative risk for bladder cancer for statistically significant RR, OR or SMR strictly above 2 and equal to or below 5;
- and a very high relative risk for bladder cancer for statistically significant RR, OR or SMR strictly above 5.

Consulted bibliographical databases included: Medline (National Library of Medicine, USA), Cochrane Library (Wiley Interscience, USA), Pascal - Institut national de l’information scientifique et technique (National scientific and technical information institute, France), National Guideline Clearinghouse (Agency for Healthcare Research and Quality, USA), Guidelines Finder (National Library for Health, USA).

We also consulted a number of websites: INRS, InVS, HAS (French National Authority for Health), Lemanissier medical library, National Institute for Health and Clinical Excellence, Scottish Intercollegiate Guidelines Network, The National Cancer Institute for Occupational Safety and Health (NIOSH) and the websites published by learned societies involved in the project.

We also used other sources of information: the bibliographical references quoted in analysed articles, recommendations (Canadian, German and American) on the screening of bladder cancer, the National Toxicology Program classification of chemical carcinogenic substances and the European Union of Dangerous Substances classification.

Concerning occupational risk factors, only publications in English and French were selected, all dating from 1950 to 1st September 2011, by using the following key words: "urinary bladder neoplasms”, “bladder neoplasm”, “bladder cancer”, “bladder tumour”, “occupational diseases”, “occupation”, "occupational exposure”, “work”, “latency”, “natural history”, “treatment”, “sensitivity”, specificity”, “screening” and all key words concerning bladder carcinogens and activities potentially exposing to bladder carcinogens (“aromatic amines”, “polycyclic aromatic hydrocarbons”, “nitrosamines”, “rubber industry”, “dye industry”, “tannery”, “leather”, “leather industry”, “hairdressers”, “chemical industry”, “chemical plants”, “laboratory”, “research laboratory”, “printing industry”, “aluminium”, “aluminium reduction plant”, “aluminium production”, “aluminium industry”, “coal tar pitch”, “Söderberg”, “foundry”, “steel”, “iron”, “tar”, “tar distillation”, “distillery”, “creosote”, “calcium carbide”, “coal gasification”, “coal”, “gas workers”, “shale oils”, “carbon black”, “carbon”, “electrode”, “electrode manufacturing”, “coke”, “coke industry”, “coke production”, “roofer”, “waterproofer”, “bituminen”, “diesel”, “diesel exhausts”, “diesel engine exhausts”, “gasoline”, “gasoline engine exhausts”, “paint”, “painter”, “varnish” “lacquer”.). Concerning bladder cancer screening tests, we exclusively selected publications in English and French, all dating from 1990 to 1st September 2011 by using, the key words: “screening”, “urinary tests”, “fluorescence immunocytochemistry urinary test”, “urinary cytology”, “NMP 22”, “NMP22BC test”, “Fluorescence In Situ Hybridisation”, “FISH, “Fibrinogen Degradation Products”, “Bladder Tumour Antigen”, “cytokeratin”, “fibroblast growth factor receptor”, “microsatellites”. A total of 2,278 references were obtained. The first selection of articles was based on the title and abstract to include only meta-analysis, systematic reviews, cohort studies which were "well-conducted" (i.e. taking into account confounding factors and potential dose–response relationships) and case–control studies for which occupational exposure was clearly described. A total of 682 articles was finally analysed. All consulted sources (except Canadian, German and American recommendations) contained peer-reviewed data.

A working group of 23 members was created, comprising 4 members from the SFMT (French Society for Occupational Health) designated by their own society, 2 oncologists designated by the SFC (French Cancer Society), 3 urologists designated by the AFU (French Urology Association), one member from the INRS (French Research and Safety Institute), one member from the InVS (French Institute for Public Health Surveillance), occupational physicians, general practitioners, epidemiologists, a biologist, an anatomopathologist and members of patient associations.

The first draft of proposed recommendations was read by a reading group comprised of 87 healthcare professionals (1 member from the *Direction Générale du Travail* (General Directorate for Labour), 1 member from the Caisse Nationale d’Assurance Maladie des Travailleurs Salariés, Direction des Risques Professionnels (French health insurance fund for salaried workers - Professional risks directorate), 48 occupational physicians (medical practitioners, university hospital practitioners and screening institution practitioners), 5 work and labour inspection practitioners, 12 general practitioners, 9 urologists, 5 oncologists, 7 representatives from partner social institutions and 3 research and prevention engineers), with a response rate of 84.6%. Readers were asked to critically comment the working group’s argumentation and to grade their agreement with its formulated recommendations (1 in the case of total disagreement, 9 in the case of total agreement). The draft was therefore revised where appropriate by the working group according to readers’ comments. The final version of the recommendation argumentation was analysed by the HAS good professional practice recommendation committee and the HAS scientific college.

Since no human being was involved in this study, no Ethics Committee or Institutional Review Board approval was necessary. For the same reason, no written informed consent was necessary.

## Results

Table 2 and Table 3 summarise the results of analysis of the scientific literature concerning identification of carcinogenic risk groups (Table 2 defines categories of workers to be discussed for inclusion in a targeted bladder cancer screening programme. Table 3 defines categories of workers for whom high exposure to occupational bladder carcinogens has generally been established without specific published epidemiological studies on bladder cancer risk in these groups of workers). We have chosen to list the occupations or sectors of activity associated with increased bladder cancer risk, rather than the incriminated chemical agents, in order to render the table more suitable for use by occupational physicians (or general practitioners/urologists), when defining their medical surveillance strategy. As expected, risk level after analysis of the literature is very high with a high degree of proof for rubber industry workers and dye manufacturing workers. Risk level is high for plastics industry workers if exposed to 4,4’-methylene bis (chloroaniline) (MBOCA), for workers involved in the production of 4-chloro-ortho-toluidine-based pesticides, but also for textile industry (dyeing) workers and for leather and tanning industry workers. Moderate risk levels are observed for many occupations, such as hairdressers and assimilated professions, workers exposed to coal combustion soot or painters. Surprisingly, for certain workers, such as road surfacing workers, workers using coal-tar creosotes, calcium carbide production workers, shale oil extraction workers or coke manufacturing workers, there was insufficient data to establish risk level after analysis of the literature, even if high exposure to occupational bladder carcinogens has generally been established for these workers.

**Table 2:** **Worker categories to be discussed for inclusion in a targeted bladder cancer screening programme**

Targeted occupational group (main references)	Position	Level of risk after analysis of the literature*	Period of exposure in France
Rubber industry workers (level of proof 1) [14–27]	- rubber production workers, using 4-aminobiphenyl and/or β-naphtylamine and its salts and/or MBOCA	VERY HIGH	Generally before 1989
			Before 1950 for subjects exclusively exposed to β-naphtylamine and its salts
	Principle exposing positions in rubber and tyre manufacturing include weighing and mixing, finishing and storage, baking or vulcanisation exposing workers to PAHs and nitrosamines.		Before 1970 for subjects exclusively exposed to 4-aminobiphenyl
			However: harmful residual carcinogenic substances (PAHs and nitrosamines in particular) remained in use in the rubber industry beyond the 1980s.
Dye manufacturing workers (level of proof 1) [28–46]	- workers in the production of benzidine and/or benzidine-derived and/or auramine-derived and/or ortho-toluidine-derived and/or magenta-derived and/or o-dianisidine-derived and/or o-tolidine-derived and/or 3,3'-dichlorobenzidine-derived and/or 2-methoxy 5 methylaniline-derived colouring agents	VERY HIGH	Generally before 1989
			Before 1980 for subjects exclusively exposed to benzidine
			Before 1990 for subjects exclusively exposed to ortho-toluidine
	- subjects working in production workshops where the aforementioned products are used		
	Principle exposing activities include: preparation and weighing activities; pigment, colouring agent, paint and varnish manufacturing; quality control, sampling, laboratory and cleaning positions.		
Textile industry (dyeing) workers (level of proof 3 to 4) [47–50]	- yarn dyed fabric workers	HIGH	Before 1970
			However: harmful residual carcinogenic substances (PAHs and nitrosamines in particular) remained in use in the textile dyeing industry beyond the 1970s.
Leather and tanning industry workers (level of proof 3) [51–56]	- leather shoe and/or boot manufacturing workers	HIGH	Before 1990
	- leather shoe and/or boot repair workers		
	- tanning, leather goods, leather processing workers		
Targeted occupational group (main references)	Position	Level of risk after analysis of the literature*	Period of exposure
Plastics industry workers, if exposed to 4,4’-methylene bis (chloroaniline) (MBOCA) [57–69]	- workers using epoxy and polyurethane resin hardening agents	HIGH	Since the 1950s and still in use today
	- subjects working in workshops where epoxy and polyurethane resin hardening agents are used		
Workers involved in the production of 4-chloro-ortho-toluidine-based pesticides (level of proof 2) [70]	- chlordimeform production workers	HIGH	Before 1986
	- subjects working in workshops where chlordimeform has been produced		
Workers involved in aluminium production (level of proof 1) [71–89]	aluminium production workers having used the Søderberg process	HIGH	Before 1989
Textile industry (weaving) workers (level of proof 2 to 3) [47–50]	- workers weaving fibre into fabric	MODERATE	Before 2003
Hairdressers and assimilated professions (level of proof 2) [90–98]	- hairdressers	MODERATE	Before 1980
	- barbers		
	- beauticians		
Workers involved in the plastics industry in general [57–69]	-production agents (after detailed assessment of specific exposure to carcinogenic agents, PAHs in particular)	MODERATE	To the present day
Chemical and pharmaceutical industry workers [99–107]	-production agents (after detailed assessment of specific exposure to carcinogenic agents)	MODERATE	To the present day
Printing industry workers [108–111]	- ink manufacturing	MODERATE	Before 1970
	- printers		
Targeted occupational group (main references)	Position	Level of risk after analysis of the literature*	Period of exposure
Iron and/or steel foundry workers [112–120]	- iron and/or steel production workers (casting and/or knockout in particular)	MODERATE	To the present day
Workers exposed to coal combustion soot [95, 121]	- chimney sweeps	MODERATE	To the present day
	- coal fire boiler room workers and those having manufactured coal nuts		Before 2007
Workers involved in coal gas production [122–124]	- coal gas production workers	MODERATE	Before 1970
Roof waterproofing work [125–128]	- roofers	MODERATE	To the present day
	- waterproofing workers		
Workers exposed to diesel engine exhaust fumes [129–132]	- professional diesel engine vehicle drivers: (heavy-goods vehicle drivers, public transport drivers, taxi drivers, work site vehicle drivers, diesel locomotive drivers)	MODERATE	To the present day
	- motor vehicle mechanics		
	- automobile control technicians (MOT/inspection)		
	- 2-wheeled vehicle delivery drivers		
	- police officers		
	- tollbooth attendants		
Metalworkers or fitters exposed to cutting oils and fluids [133–140]	- metal machining workers	MODERATE	To the present day
	- tool-dressers		
	- adjusters (tasks: machining, cutting, welding, degreasing, maintenance/trimming)		
Painters [141–149]	- painters	MODERATE	Before 1970 (after 1980 in the case of use of epoxy or polyurethane anti-corrosion paints)
Transport vehicle repair and construction [95, 139, 150, 151]	- tool-dressers	MODERATE	To the present day
	- adjusters		
Dry cleaning workers [152–159]	dry cleaning workers	MODERATE	To the present day
Wine growers [160]	- use of arsenic-based pesticides	MODERATE	Before 2001

* VERY HIGH relative risk for relative risks (RR), Odds ratios (OR) or Standardised Mortality Ratios (SMR) observed in the scientific literature strictly above 5; HIGH relative risk for RR, OR or SMR strictly above 2 and equal to or below 5 and MODERATE relative risk for RR, OR or SMR strictly above 1 and equal to or below 2.

**Table 3:** **Categories of workers for whom high exposure to occupational bladder carcinogens has generally been established without specific epidemiological studies on the risk of bladder cancer in these groups of workers**

Targeted occupational group (main references)	Position	Level of risk after analysis of the literature*	Period of exposure
**Research laboratory workers** [161]**-**[167]	Genetic engineering, nuclear biology, mutagenesis and cancerogenesis laboratories, weighing activities, use of reagents and synthetic intermediates*	**Insufficient data to establish risk level**	To the present day
**Road surfacing workers** [125]**-**[128]	Asphalt spreaders, gritters, compactors	**Insufficient data to establish risk level**	Up to the late 1980s
**Workers using coal-tar creosotes** [112]**,**[168]**-**[170]	Workers conducting specific wood treatment activities	**Insufficient data to establish risk level**	To the present day
**Calcium carbide production workers** [171]	Production workers*	**Insufficient data to establish risk level**	Up to the mid 2000s
**Shale oil extraction workers** [172]	Shale oil extraction workers	**Insufficient data to establish risk level**	To the present day
**Carbon black production workers** [50]**,**[173]**-**[176]	Manufacturing workers*	**Insufficient data to establish risk level**	To the present day
**Carbon electrode manufacturing workers** [177]**-**[179]	Manufacturing workers*	**Insufficient data to establish risk level**	To the present day
**Coke manufacturing workers** [125]**-**[128]**,**[180]**-**[186]	Cokers	**Insufficient data to establish risk level**	To the present day
**Carbon nut industry** [187]	Manufacturing workers*	**Insufficient data to establish risk level**	To the present day
**Carbon disc manufacturing** [188]	Manufacturing* and maintenance workers	**Insufficient data to establish risk level**	To the present day
**Clay pigeon manufacturing** [188]	Manufacturing workers*	**Insufficient data to establish risk level**	To the present day
**Cement oven repair** [188]	Manufacturing workers*	**Insufficient data to establish risk level**	To the present day
**Work on water conveyance conducts coated with varnish containing HAP** [188]	Technicians/repair workers*	**Insufficient data to establish risk level**	To the present day
**Glazing in aluminium foundries** [188]	Glazers	**Insufficient data to establish risk level**	To the present day

*After detailed assessment of specific exposure to carcinogenic agents.

Table 4 summarises the results of the performance of proposed and marketed urinary tests for diagnosing bladder cancer, their availability, acceptability, adverse effects and cost. In this table, data including confidence intervals for sensitivity and specificity of urinary cytology, FISH, ImmunoCyt and NMP22 for the detection and follow-up of bladder cancer, result from a systematic review conducted in 2010 by the National Institute for Health Research (NIHR), and published as part of its Health Technology Assessment (HTA) programme [189]. Among the marketed urinary tests likely to be used in targeted bladder cancer screening, the fluorescence immunocytochemistry urinary test is the one that offers the best sensitivity all tumour stages and grades combined (evaluated at 84% [IC 95%, 77–91]) [189], whereas urinary cytology offers the best specificity all tumour stages and grades combined (above 90%) [189–197].

**Table 4:** **Proposed and marketed urinary tests for bladder cancer screening: summary of performance, availability, acceptability, adverse effects**

Test/dosage	Sensitivity	Specificity	Main references
**Detection of haematuria using a reactive test strip**	46 to 74% for one test, If test repeated over several days: 90 to 95%	51 to 84%	[190, 198–202]
**Urinary cytology**	▪ For all tumour grades and stages: 44% [CI 95%, 38–51] [203]	▪ For all tumour grades and stages: **96% [CI 95%, 94–98]**[203]	[191–197, 203, 204]
	▪ **For Carcinoma in situ (Cis) 70 to 90%**	▪ For Cis: 90%	
**NMP22BC test**	▪ For all tumour grades and stages: 65% [CI 95%, 50–80] [203]	▪ For all tumour grades and stages: 81% [CI 95%, 50–85] [203]	[197, 203–209]
	- **NMIBC: 81.8%**		
	- MIBC: 57.1%		
	- **Grade G1: 83.9%**		
	**-** Grade G3: 62.5%		
**Fluorescence immunocytochemistry (ImmunoCyt™/uCyt + ™)**	▪ For all tumour grades and stages: 84% [CI 95%, 77–91] [203]	▪ For all tumour grades and stages: 75% [CI 95%, 68–83] [203]	[189, 203, 210–213]
**FISH (Fluorescence In Situ Hybridisation) UroVysion™** **Kit**	▪ For all tumour grades and stages: 76% [CI 95%, 65–84] [203]	▪ For all tumour grades and stages: 75% [CI 95%, 78–92] [203]	[189, 191, 192, 203, 214–218]
	▪ **For Cis and G3: >95%**		

In studies focusing on the sensitivity and the specificity of the urinary tests used combined, sensitivity is above that of any one test used alone (whereas specificity is, of course, lower than that of one test used alone). The combination of urinary cytology and fluorescence immunocytochemistry considerably increases sensitivity (mean: 85%), compared to urinary cytology alone, in particular for the detection of low-grade tumours. The average specificity of the two tests combined is 70% [192, 195, 197, 209].

The high cost of fluorescence immunocytochemistry and its limited availability (laboratory tests cannot be used during medical consultations and are performed by only a few French laboratories) mean that it is not the choice test for monitoring bladder carcinogen-exposed workers within a context of targeted screening of bladder cancer of occupational origin.

We conducted performance simulations for the different screening tests proposed (and combinations thereof), on a population of 100,000 male subjects aged from 50 to 74 years, based on their degree of exposure to bladder carcinogens (refer to Additional file 1: Table S1). Bladder cancer incidence in this population was 54.6 cases/100,000. In the absence of sufficient data, the sensitivity and specificity of test combinations were calculated as if the test had been conducted independently. The NMP22BC test, exclusive haematuria testing and combinations of urine cytology with, respectively, the NMP22BC test and haematuria test, generated an extremely high proportion of false positive results (around 20,000 false positives for 100,000 subjects, in each high-risk group category). If we extrapolate these results to a population of 750,000 subjects concerned by potential targeted screening in France (subjects at a high and very high risk of bladder cancer), the number of false positives likely to be generated by these tests would be 300,000. Urinary cytology used alone would generate 8,000 false positives for 100,000 high or very high risk subjects which, in our population of 750,000 subjects, corresponds to 60,000 false positive results. Although very high, this figure is well below the 300,000 observed with the previously described tests.

The sensitivity of urinary cytology alone is mediocre all stages combined (mean: 44%, see Table 4), hence generating a large number of false negatives. Nevertheless, the sensitivity of this technique is the best for high grades (mean: 80%, see Table 4), which require early diagnosis since late-stage cancers are of very poor prognosis, hence rendering it the choice option for first line targeted screening strategies.

## Discussion

In 2005, a panel of international experts (International Consensus Panel on cytology and bladder tumour markers) concluded that individual screening in high-risk patients (smokers, occupationally-exposed subjects, subjects with a genetic predisposition) could be considered using, in particular, urinary makers associated or not with conventional cytology. In this indication, although no currently marketed tumour markers appear suitable for replacing cytoscopy, tests such as fluorescence immunocytochemistry, combined with cytology, microsatellites or FISH (Fluorescence In Situ Hybridisation) are of genuine interest, offering high sensitivity and a negative predictive value of 95%, hence avoiding unnecessary cytoscopies [13].

The main diagnostic limitation of urinary markers is their specificity, which is lower than that of conventional cytology and behind a substantial number of false positive results; they are also costly. In certain studies, the performance of the various tests likely to be used for bladder cancer screening were compared on the same patient: for each comparison, urinary cytology offered lower sensitivity, all grades combined, than the marker with which it was compared, whilst offering higher sensitivity for high-grade tumours and higher specificity whatever the grade.

Since several studies on bladder cancer screening tools are currently underway, the present recommendations will require to be reassessed according to results of new and ongoing studies.

The various aforementioned observations led the French team to put forward the following recommendations:

- The high cost of fluorescence immunocytochemistry and its limited availability (laboratory tests cannot be used during medical consultations and are performed by only a few French laboratories) mean that it is not the choice test for monitoring bladder carcinogen-exposed workers within a context of targeted screening of bladder cancer of occupational origin.
- Given its poor performance, in terms of both sensitivity and specificity, it is recommended to avoid the exclusive detection of microscopic haematuria using reactive urinary test strips during specific follow-up consultations for targeted screening of subjects currently or previously occupationally exposed to carcinogens.
- Repeated detection of microscopic haematuria using reactive urinary strips (daily test over 5 days, then weekly test over 51 weeks or daily test over 14 days then, in the absence of haematuria, daily test over 14 days - 9 months later) offers good sensitivity [194]. However, the constraints involved in its implementation (urinary strip packaging, uncertainty on user compliance) do not enable it to be considered as a choice examination within the context of the follow-up of workers exposed to bladder carcinogens for the targeted screening of bladder cancer of occupational origin.
- Urinary cytology (or urinary cytodiagnosis), the aim of which is to detect tumour cells originating from bladder or urinary tract cancer and desquamating in urine, is the urinary test with the best specificity (for all tumour grades and stages - on average, above 90%, see Table 4), and with the best sensitivity for high-grade tumours, requiring urgent medical care, hence its first-line use.
- According to current knowledge, the combination of urinary cytology and urinary tests such as the NMP22BC test cannot be recommended within targeted screening procedures.

### Proposal for the medical follow-up of workers currently or previously exposed to bladder carcinogens

In populations presenting with a high risk of bladder cancer subsequent to occupational exposure justifying targeted screening, screening tests are recommended 20 years after the start of exposure to the bladder carcinogen (GRADE B recommendation, “Scientific proof presumed”).

Urinary cytology among subjects currently or previously exposed to bladder carcinogens is recommended for subjects included in a targeted screening programme (expert consensus).

A 6-month periodicity is recommended for conducting targeted bladder cancer screening tests among subjects currently or previously occupationally exposed to bladder carcinogens (expert consensus).

The proposed medical surveillance protocol accounts for the performance of the various screening tests (specificity/sensitivity) and, in particular, the expected number of false positive results for the monitored population. This protocol is summarised in the following algorithm (Table 5) (expert consensus):

- RECOMMENDED (in all cases): for groups of workers with a very high risk of bladder cancer i.e. RR > 5 (see Table 2), or professions with documented high exposure levels (Table 3), with an exposure duration equal to or in excess of 1 year;
- PROPOSED (for discussion on a case-by-case basis):
  - for groups of workers with a very high risk of bladder cancer (see Table 2) or professions with documented high exposure levels (Table 3), with an exposure duration of less than 1 year;
  - for groups of workers with a high risk of bladder cancer (Table 2) with an exposure duration equal to or in excess of 1 year;
- NOT RECOMMENDED (in view of the performance of currently available tests) for groups of workers with a moderate risk of bladder cancer (Table 2), and for groups of workers with a high risk of bladder cancer (Table 2), with an exposure duration of less than 1 year.

**Table 5:** **Algorithm summarising the recommended medico-professional follow-up of workers currently or previously exposed to carcinogenic substances for the bladder**

Risk level for the professional group	Group of workers with a VERY HIGH risk (RR or OR or SMR > 5) or professions with documented high exposure levels		Group of workers with a HIGH risk (2< RR or OR or SMR ≤5)		Group of workers with a MODERATE risk (1< RR or OR or SMR ≤2)
Exposure duration	≥ 1 year	< 1 year	≥ 1 year	< 1 year	
Follow-up	RECOMMENDED	PROPOSED		NOT RECOMMENDED (in view of the performance of currently available tests)	
Minimum latency period after the start of exposure	20 years				
Proposed first line, then 6-monthly test	Urinary cytology				

Subsequent to the compilation of these recommendations, a few new articles have been published [219]. Indeed, authors analysed the performance of FISH (Fluorescence In Situ Hybridisation) in combination with NMP22 for bladder cancer screening in this population. They observed that the combination of these 2 tests detected more cases than cytology alone, at the expense of a lower specificity, and concluded that it cannot be recommended to apply these markers for screening in asymptomatic workers, given that the increase in sensitivity is not balanced by the high cost of FISH and the false-positive results obtained by NMP22. Another recent study on occupational urinary tract cancers in Great Britain described the same types of occupational exposure associated with bladder cancer as those compiled in our own study [220].

## Conclusion

Occupational cancer prevention relies first and foremost on primary prevention, in other words, not only the identification of carcinogenic substances present in the working environment, but also on the evaluation of individual and collective exposure, hence enabling the implementation of measures aimed at eliminating/controlling these substances. Such prevention implies accurate risk assessment, taking into account current scientific knowledge on modes of action, dose-effect relationships and on the potential existence of an effect threshold, in order to enable the reduction and the traceability of occupational exposure. Such traceability must offer workers the benefits of improved knowledge in terms of information on toxicity and medical follow-up. Certain authors have even established a guideline to assess occupational bladder cancer risk. For example, a German research group has provided a multilingual questionnaire on occupational and further bladder cancer risk factors [221]. This questionnaire asks for relevant medical information, for the occupational history since leaving school and for intensity and frequency of certain occupational and non-occupational risk factors. In another study, the authors established a guideline specifically evaluating occupational bladder cancer risk for compensation [222].

Even if no study has relied on a sufficient sample of individuals to assess the relevance of bladder cancer screening in populations at risk, we are convinced of the necessity to implement recommendations that rely on analysis of the scientific literature to provide a more rational definition of medical surveillance modalities for subjects currently or previously exposed to bladder carcinogens.

Nevertheless, an evaluation of the benefits of this targeted screening strategy for bladder cancer is recommended. Given ongoing research on the different urinary markers for potential use in targeted bladder cancer screening, analysis of the feasibility and the performance of a targeted bladder screening programme combining urinary cytology and other urinary markers is recommended within a 5-year timescale.

## Authors’ information

“RecoCancerProf” Working Group: *Yves ALLORY, Service d’Anatomie Pathologique, Centre Hospitalier Henri Mondor, Créteil (94) (yves.allory@hmn.aphp.fr), Jérôme BEAUJARD, La Fare les Oliviers (13), (Jerome.BEAUJARD@wanadoo.fr), Dominique BESSETTE, INCa, Boulogne (92) (dbessette@institutcancer.fr), Patrick BROCHARD, Service de Santé au Travail et Pathologie Professionnelle, Centre Hospitalier de Bordeaux (33) (patrick.brochard@chu-bordeaux.fr), Michèle CHATAIGNIER, pour l’Association des Accidentés de la Vie, Bressuire (79) (michele.erisse@orange.fr), Jacqueline CLAVEL, Institut national de la santé et de la recherche médicale (INSERM), Villejuif (94) (jacqueline.clavel@inserm.fr), Françoise CONSO, Membre de la SFMT, Paris (75) (francoise.conso@parisdescartes.fr), Françoise FAUPIN, Suresnes (92) (Francoise.faupin@acms.asso.fr), Jean-François GEHANNO, Service de médecine et santé au travail, Centre Hospitalier de Rouen (76) (JF.Gehanno@chu-rouen.fr), Philippe GRIPON, Renault Trucks, Blainville sur Orne (14) (Philippe.gripon@renault-trucks.com), Marine GROSS-GOUPIL, Service d’oncologie, Centre Hospitalier de Bordeaux (33) (marine.gross-goupil@chu-bordeaux.fr), Laurent GUY, Service d’urologie, Hôpital G. Montpied, Clermont-Ferrand (63) (lguy@chu-clermontferrand.fr), Michel HERY, INRS, Vandœuvre (54) (michel.hery@inrs.fr), Nadine HOUEDE, Service d’Oncologie, Institut Bergonié, Bordeaux (33) (houede@bergonie.org), Ellen IMBERNON, InVS, Saint-Maurice (94) (e.imbernon@invs.sante.fr), Danièle LUCE, InVS, Saint-Maurice (94) (daniele.luce@inserm.fr), Christophe PARIS, Service de médecine et santé au travail, membre de la SFMT, Centre Hospitalier de Nancy (54) (christophe.paris@inserm.fr), Christian PFISTER, Service d’Urologie, Centre Hospitalier de Rouen (76) (christian.pfister@chu-rouen.fr), François RADVANYI, Service de Biologie, Centre Hospitalier Henri Mondor, Créteil (94) (Francois.Radvanyi@curie.fr), Alain RAVAUD, Service d’Oncologie, membre de la SFC, Centre Hospitalier de Bordeaux (33) (alain.ravaud@chu-bordeaux.fr), Morgan ROUPRET, Service d’Urologie, Hôpital Pitié-Salpétrière (AP-HP), Université Pierre et Marie Curie, Paris (75) (mroupret@gmail.com), Marcel RUETSCH, Dessenheim (68) (dr.m.ruetsch.mg@wanadoo.fr) and Marie-Paule VIGOUROUX, pour la Ligue Nationale contre le cancer, Plescop (56) (mpvigx@wanadoo.fr).

## Electronic supplementary material

Additional file 1: Table S1: Simulated performance of proposed screening tests (and combinations). (DOC 56 KB)

Below are the links to the authors’ original submitted files for images.

Authors’ original file for figure 1
