# Supplementary material for: Medical follow-up for workers exposed to bladder carcinogens: the French evidence-based and pragmatic statement
Source: BMC Public Health. 2014 Nov 6;14:1155. doi: 10.1186/1471-2458-14-1155 (PMC4230399; doi:10.1186/1471-2458-14-1155)
Supplement: Supplementary file 1 — Additional file 1: Table S1: Simulated performance of proposed screening tests (and combinations). (DOC 56 KB) [file 12889_2014_7220_MOESM1_ESM.doc]

**Additional file 1: Table S1 Simulated performance of proposed screening tests (and combinations).**

| **Proposed Test**  (Se) ; (Sp) | **Number of men: 100 000**  **Age: between 50 and 74 years**  **Incidence rate of bladder cancer in this population: 54.9/100000** | | |
| --- | --- | --- | --- |
|  | **Very high risk professional group (RR=7.5)**  **N exp*= 410 cases** | **High risk professional group**  **(RR=3.5)**  **N exp*= 191 cases** | **Moderate risk professional group**  **(RR=1.5)**  **N exp*= 82 cases** |
| **Urinary cytology all stages combined**  (Se=0.44 ; Sp=0.96) | TP: n=180  FN: n=229  FP: n=3984  PPV: 4.33% | TP: n=84  FN: n=107  FP: n=3992  PPV: 2.06% | TP: n=36  FN: n=46  FP: n=3997  PPV: 0.89% |
| **Urinary cytology for high tumour grades**  (Se=80%) | **Nexp HG**=205**  TP: n=164  FN: n=41  FP: n=3992 | **Nexp HG**=84**  TP: n=76  FN: n=19  FP: n=3996 | **Nexp HG**=36**  TP: n=33  FN: n=8  FP: n=3998 |
| **NMP22BC**  (Se=0.65 ; Sp=0.81) | TP: n=266  FN: n=144  **FP: n=18922**  PPV: 2.13% | TP: n=124  FN: n= 67  **FP: n=18964**  PPV: 1.0% | TP: n=53  FN: n=29  **FP: n=18985**  PPV: 0.43% |
| **Haematuria detection only**  (Se=0.50 ; Sp=0.80) | TP: n=205  FN: n=205  **FP: n=19918**  PPV: 0.20% | TP: n=95  FN: n=95  **FP: n=19961**  PPV: n=0.09% | TP: n=41  FN: n=41  **FP: n=19983**  PPV: 0.04% |
| **Combination of cytology+haematuria detection*****  (Se=0.72 ; Sp=0.77) | TP: n=295  FN: n=115  **FP: n=23105**  PPV: 1.26% | TP: n=138  FN: n=54  **FP: n=23156**  PPV: 0.59% | TP: n=59  FN: n=23  **FP: n=23181**  PPV: 0.25% |
| **Combination of cytology + NMP22BC*****  (Se=0.80 ; Sp=0.78) | TP: n=329  FN: n=80  **FP: n=22149**  PPV: 1.46% | TP: n=154  FN: n=37  **FP: n=22197**  PPV: 0.69% | TP: n=66  FN: n=16  **FP: n=22222**  PPV: 0.30% |

*Nexp: expected number of bladder cancer cases

** Nexp HG: expected number of high grade bladder cancer cases

*** Positive test if at least one of the 2 tests is positive in the case of test combinations.

Captions: True positives=TP; False positives=FP ; True negatives=TN ; Positive Predictive Value=PPV ; Sensitivity=Se ; Specificity=Sp (in the absence of data, sensitivities and specificities of test combinations were calculated based on the supposition that they were independent).
