# Supplementary figures and images for: Medical follow-up for workers exposed to bladder carcinogens: the French evidence-based and pragmatic statement
Source: BMC Public Health. 2014 Nov 6;14:1155. doi: 10.1186/1471-2458-14-1155 (PMC4230399; doi:10.1186/1471-2458-14-1155)

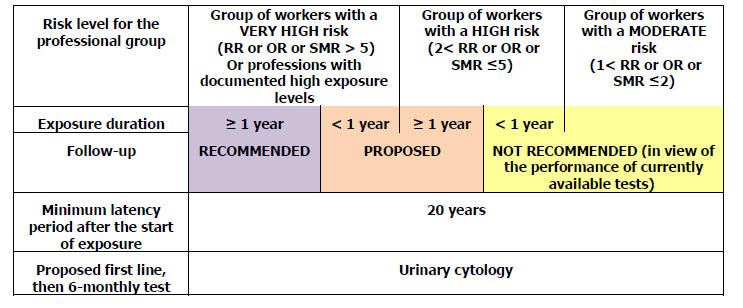

Supplement: Supplementary file 2 — Authors’ original file for figure 1 [file 12889_2014_7220_MOESM2_ESM.tiff]
